# Race, Insurance, and Sex-Based Disparities in Access to High-Volume Centers for Pancreatectomy

**DOI:** 10.1245/s10434-022-13032-8

**Published:** 2023-01-02

**Authors:** Catherine G. Williamson, Shayan Ebrahimian, Sara Sakowitz, Esteban Aguayo, Elsa Kronen, Timothy R. Donahue, Peyman Benharash

**Affiliations:** 1 Cardiovascular Outcomes Research Laboratories (CORELAB), UCLA Center for Health Sciences, David Geffen School of Medicine at UCLA, Los Angeles, CA USA; 2 Department of Surgery, David Geffen School of Medicine at UCLA, Los Angeles, CA USA

## Abstract

**Background:**

With a large body of literature demonstrating positive volume-outcome relationships for most major operations, minimum volume requirements have been suggested for concentration of cases to high-volume centers (HVCs). However, data are limited regarding disparities in access to these hospitals for pancreatectomy patients.

**Methods:**

The 2005–2018 National Inpatient Sample (NIS) was queried for all elective adult hospitalizations for pancreatectomy. Hospitals performing more than 20 annual cases were classified as HVCs. Mixed-multivariable regression models were developed to characterize the impact of demographic factors and case volume on outcomes of interest.

**Results:**

Of an estimated 127,527 hospitalizations, 79.8% occurred at HVCs. Patients at these centers were more frequently white (79.0 vs 70.8%; *p* < 0.001), privately insured (39.4 vs 34.2%; *p* < 0.001), and within the highest income quartile (30.5 vs 25.0%; *p* < 0.001). Adjusted analysis showed that operations performed at HVCs were associated with reduced odds of in-hospital mortality (adjusted odds ratio [AOR], 0.43; 95% confidence interval [CI], 0.34–0.55), increased odds of discharge to home (AOR, 1.17; 95% CI, 1.04–1.30), shorter hospital stay (β, −0.81 days; 95% CI, −1.2 to −0.40 days), but similar costs. Patients who were female (AOR, 0.88; 95% CI, 0.79–0.98), non-white (black: AOR, 0.66; 95% CI, 0.59–0.75; Hispanic: AOR, 0.56; 95% CI, 0.47–0.66; reference, white), insured by Medicaid (AOR, 0.63; 95% CI, 0.56–0.72; reference, private), and within the lowest income quartile (AOR, 0.73; 95% CI, 0.59–0.90; reference, highest) had decreased odds of treatment at an HVC.

**Conclusions:**

For those undergoing pancreatectomies, HVCs realize superior clinical outcomes but treat lower proportions of female, non-white, and Medicaid populations. These findings may have implications for improving access to high-quality centers.

A large body of literature has characterized the relationship between hospital surgical caseload and outcomes of pancreatic resections for cancer, with high-volume centers (HVCs) exhibiting significantly lower perioperative mortality and shorter inpatient stays.^1^ Based on these findings, many have proposed centralization of care and enforcement of minimum-volume standards to improve overall survival.^1^ The use of such standards is particularly relevant because pancreatic cancer is projected to be the second leading cause of malignancy-associated deaths by 2030.^2^ However, the concept of centralization has been criticized by many with concerns regarding its contributions toward reduced access to care, particularly for the socioeconomically disadvantaged.^3–7^

Socioeconomic disparities have long been implicated in resection rates and outcomes of operations for pancreatic cancer.^8–11^ Proposed explanations for these disparities include the fact that disadvantaged populations more commonly receive care at low-volume and lower-quality hospitals.^12^ In a study of pancreatic malignancy resections from 1996 to 2006, patients at low-volume hospitals were significantly more likely to be black, have Medicaid or no insurance, and come from lower-income areas than patients at HVCs.^3^ Despite the acceptance of hospital volume-outcome associations in surgical care for pancreatectomy, studies evaluating the impact of centralization on existing socioeconomic disparities remain outdated and limited in size and generalizability.^3^

The current study used a national database to characterize disparities in treatment at HVCs among patients undergoing pancreatectomy. We further examined in-hospital clinical outcomes and expenditures associated with hospital pancreatectomy volume status. We hypothesized that access to HVCs would be limited for under-insured, non-white, low-income, and female populations.

## Methods

In this retrospective cohort study, the 2005–2018 National Inpatient Sample (NIS) was used to identify all elective adult hospitalizations for cancer-related pancreatectomy.^13^ The NIS is the largest publicly available all-payer inpatient database in the United States and provides accurate estimates for 97% of annual hospitalizations using survey-weighted methodology. In 2012, the NIS methodology changed from sampling all discharges at 20% of hospitals to 20% of cases at participating facilities. Therefore, the NIS uses trend and discharge weights to account for clustering and provides national estimates accordingly.

Relevant patient, operative, and hospital characteristics were extracted using the *International Classification of Diseases, Ninth and Tenth Revisions* (ICD-9/10) codes and the Healthcare Cost and Utilization Project data dictionary. Variables such as insurance status, race, income, and sex were defined according to the NIS data dictionary. Income is classified by NIS as an estimated median household income for the respective patient zip code. Pancreatectomy for cancer was coded as has been previously described.^14^ Patients with missing information for mortality, income, insurance, race, or sex were excluded from analysis (10.8%).

Centers were classified as low-volume centers (LVCs), medium-volume centers, or HVCs based on the annual case volume of pancreatectomy, with cutoffs at 2 annual cases for LVCs and 20 annual cases for HVCs, as established by the Leapfrog Initiative.^15–17^ The Elixhauser Comorbidity Index, a previously validated composite score using 30 chronic conditions, was used to estimate the burden of comorbidities numerically.^18^ Hospitalization costs, calculated using center-specific cost-to-charge ratios, were inflation-adjusted to the 2018 Personal Healthcare-Hospital Index.^19^

Categorical variables are reported as frequency (%) and continuous variables as means with standard deviation. Covariates with a non-normal distribution are reported as medians and interquartile ranges (IQRs). The Pearson’s chi-square test was used to compare categorical variables. Means were compared using the adjusted Wald test, and medians were compared using the Mann-Whitney *U* test.

The primary outcome of interest was treatment at an HVC, but the association of HVC status with mortality, complications, discharge disposition, and resource use also was examined. Multivariable adjusted models were generated with variable selection guided by elastic net regularization.^20^ This methodology provides penalized selection of variables to maximize out-of-sample validity and reduce the potential for overfitting.

The models were tested using 10-fold cross-validation.^21^ Variables ultimately chosen for the model for treatment at an HVC included patient race, age, sex, income, insurance, indication for operation, Elixhauser comorbidity index, and calendar year. The models for other end points included all the aforementioned variables as well as hospital region, hospital teaching status, and hospital pancreatectomy volume. These outcomes are reported as adjusted odds ratios (AORs) for dichotomous outcomes and beta coefficients (β) for continuous outcomes, with 95% confidence intervals (95% CI) for both.

An alpha lower than 0.05 was considered statistically significant. All statistical analyses were performed using Stata 16.1, College Station, Texas. This study was deemed exempt from full review by the Institutional Review Board at the University of California, Los Angeles.

## Results

### Baseline Characteristics

Of the estimated 127,527 hospitalizations included for analysis, 101,815 (79.8%) occurred at an HVC. Hospitals classified as HVC managed a median of 33 cases (IQR, 18–69 cases) per year, whereas those classified as medium-volume centers managed 3 cases (IQR, 2–5 cases) annually, and those classified as LVC managed 1 case (IQR, 1–1) per year.

Compared with the patients treated at LVCs, those treated at HVCs were more frequently white (79.0 vs 70.8%; *p* < 0.001), privately insured (39.4 vs 34.2%; *p* < 0.001), and within the highest income quartile (30.5 vs 25.0%; *p* < 0.001) (Table 1). Those treated at HVCs were similar in age to those treated at LVCs (65.1 vs 65.2 years; *p* = 0.72), but exhibited a lower comorbidity burden as measured by the Elixhauser comorbidity index (3.58 vs 3.78 points; *p* < 0.001). These patients had higher rates of malignant pancreatic neoplasms (88.9 vs 84.4%; *p* < 0.001), but lower rates of comorbidities such as chronic obstructive pulmonary disease (COPD) (12.0 vs 14.5%; *p* < 0.001), smoking status (9.32 vs 11.5%; *p* < 0.001), congestive heart failure (3.85 vs 5.27%; *p* < 0.001) and coagulopathies (4.85 vs 6.31; *p* < 0.001) (Table 1).

**Table 1:** Demographic, clinical, and outcome measures of patients undergoing pancreatectomy by hospital volume

	LVC (*n* = 25,713) *n* (%)	HVC (*n* = 101,815) *n* (%)	*p* Value
*Demographics*			
Age	65.2 ± 11.8	65.1 ± 11.5	0.72
Female sex	12,522 (48.7)	48,852 (48.0)	0.37
Elixhauser Comorbidity Score	3.78 ± 1.75	3.58 ± 1.64	<0.001
*Race*			< 0.001
White	16,653 (70.8)	71,662 (79.0)	
Black	2659 (11.3)	6966 (7.68)	
Hispanic	2494 (10.6)	5978 (6.59)	
Asian	920 (3.91)	2848 (3.14)	
Other	802 (3.40)	3288 (3.63)	
*Median household income quartile (percentile)*			< 0.001
0–24th	5581 (22.9)	18,499 (19.5)	
25–49th	6374 (26.1)	22,505 (23.7)	
50–74th	6335 (26.0)	24,992 (26.3)	
75–99th	6093 (25.0)	28,929 (30.5)	
*Insurance status*			< 0.001
Medicare	13,657 (53.2)	52,826 (52.0)	
Medicaid	2058 (8.02)	4726 (4.65)	
Private	8779 (34.2)	40,030 (39.4)	
Other	554 (2.15)	2396 (2.35)	
*Comorbidities*			
Malignant pancreatic tail neoplasm	3019 (11.7)	8463 (8.31)	< 0.001
Malignant pancreatic head neoplasm	8631 (33.6)	42,519 (41.8)	< 0.001
Malignant pancreatic neoplasm other	4273 (16.6)	15,033 (14.8)	0.004
Malignant pancreatic duct neoplasm	460 (1.79)	2203 (2.16)	0.12
Pancreatic neuroendocrine tumor	2314 (9.00)	6773 (6.65)	< 0.001
Benign pancreatic neoplasm	163 (0.63)	726 (0.71)	0.55
Cholangiocarcinoma	314 (1.22)	1233 (1.21)	0.95
Malignancy of extrahepatic ducts	2917 (11.3)	12,364 (12.1)	0.13
Malignant neoplasm of duodenum	2615 (10.2)	10,692 (10.5)	0.50
COPD	3731 (14.5)	12,256 (12.0)	< 0.001
Smoker	2966 (11.5)	9494 (9.32)	< 0.001
Diabetes	7080 (27.5)	27,476 (27.0)	0.44
Hypertension	13,496 (52.5)	52,617 (51.7)	0.34
Coronary artery disease	3,000 (11.7)	12,527 (12.3)	0.22
Congestive heart failure	1355 (5.27)	3924 (3.85)	< 0.001
Coagulopathy	1624 (6.31)	4939 (4.85)	< 0.001
Peripheral vascular disease	1032 (4.02)	3404 (3.34)	0.02
Alcohol abuse	620 (2.41)	1908 (1.87)	0.01
Liver disease	1870 (7.27)	7612 (7.47)	0.64
Chronic kidney disease	171 (0.67)	593 (0.58)	0.48
*Hospital region*			0.005
Northeast	4036 (15.7)	23,489 (23.1)	
Midwest	6179 (24.0)	22,877 (22.5)	
South	9179 (35.7)	34,416 (33.8)	
West	6318 (24.6)	21,033 (20.7)	

*HVC* high-volume center; *LVC* low-volume center

*n* (%)

### Adjusted Outcomes

In adjusted analysis, HVCs were associated with reduced odds of in-hospital mortality (AOR, 0.43; 95% CI, 0.34–0.55), lower odds of complications (AOR, 0.71; 95% CI, 0.64–0.78), increased odds of discharge to home (AOR, 1.17; 95% CI, 1.04–1.30), decreased hospital length of stay (LOS) (β, –0.81; 95% CI, –1.2 to –0.40), and similar costs (β, –$1000; 95% CI, –$730 to $2800; Table 2).

**Table 2:** Outcome measures of patients undergoing pancreatectomy by hospital volume

	Unadjusted			Adjusted	
	LVC *n* (%)	HVC *n* (%)	*p* Value	HVC^a^	95% CI
Complications (AOR)	9,889 (38.5)	32,134 (31.6)	< 0.001	0.71	0.64–0.78
Mortality (AOR)	1,169 (4.55)	2,464 (2.42)	< 0.001	0.43	0.34–0.55
Hospital stay: days (β)	12.7 ± 11.0	11.4 ± 10.1	< 0.001	–0.81	–1.2 to –0.40
Costs: $1000 (β)	42.7 ± 37.4	41.6 ± 38.5	0.19	+1.0	–0.73 to 2.8
Home discharge (AOR)	12,899 (50.2)	54,514 (53.5)	0.006	1.17	1.04–1.30

*LVC* low-volume center; *HVC* high-volume center, *AOR* adjusted odds ratio; β, beta coefficient

^a^Adjusted outcomes, with LVC as reference

### Access to HVC

**Fig. 1:**
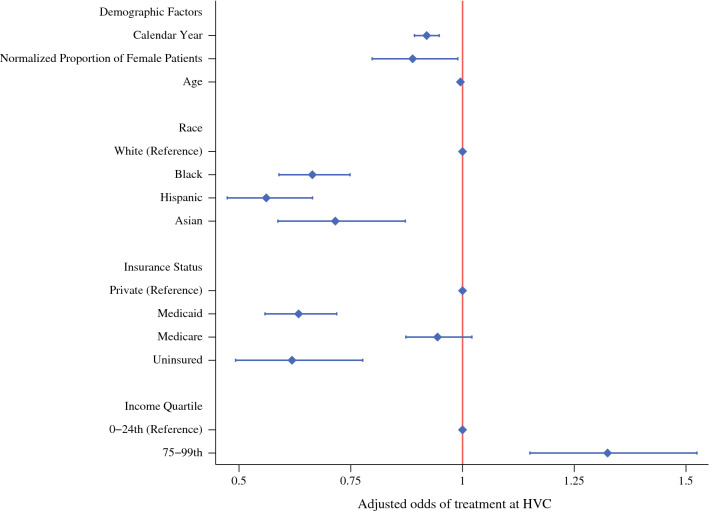
Risk-adjusted predictive model of treatment at a high-volume center

**Fig. 2:**
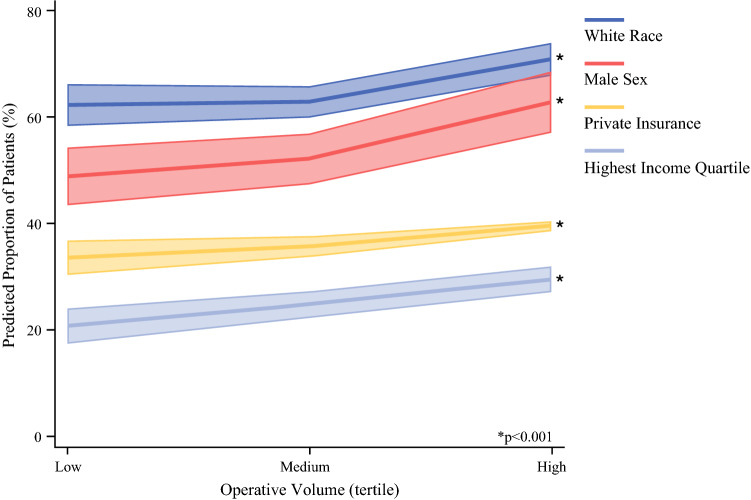
Risk-adjusted proportion of patients treated in the three categories of case volume stratified by race, sex, insurance, and income

Demographic factors associated with HVCs are shown in Fig. 1. Patients who were female (AOR, 0.88 [95% CI, 0.79–0.98]), non-white (black AOR, 0.66 [95% CI, 0.59-0.75]; Hispanic AOR, 0.56 [95% CI, 0.47–0.66]; reference, white), insured by Medicaid (AOR, 0.63 [95% CI, 0.56–0.72]; reference, private), and within the lowest income quartile (AOR, 0.73 [95% CI, 0.59–0.90]; reference, highest) had lower odds of treatment at an HVC than at an LVC. Figure 2 depicts the predicted proportion of patients treated at institutions categorized by hospital volume status and demographic factors. As exhibited in Fig. 2, insurance status, income, male sex, and race all linearly correlated with surgical volume, with minority groups comprising a smaller proportion of the patient population as case volume increased (all *p* < 0.001; Fig. 2).

**Fig. 3:**
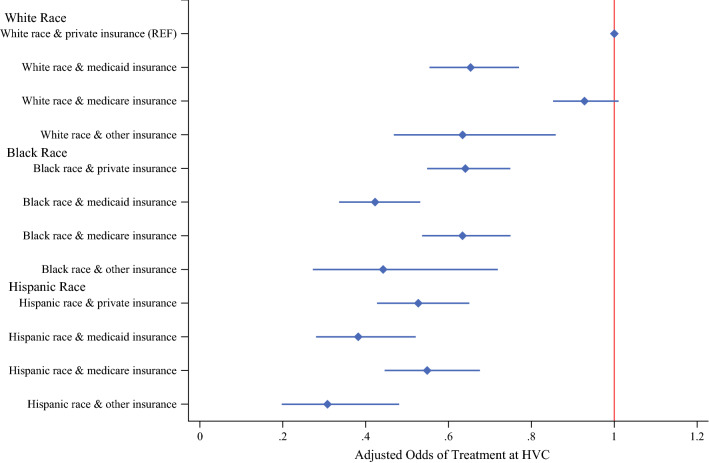
Risk-adjusted subgroup analysis of patients treated at high-volume centers stratified by both race and insurance status

In the subgroup multivariable analysis, HVC access was assessed by both race and insurance status simultaneously, as shown in Fig. 3. With white patients who had private insurance as the reference, black (AOR, 0.64; 95% CI, 0.55–0.75) and Hispanic (AOR, 0.53; 95% CI, 0.43–0.65) patients with private insurance continued to have decreased access to high-volume centers. Moreover, white patients with Medicaid (AOR, 0.65; 95% CI, 0.55–0.77) or “other” insurances including uninsured and unclassified insurance types, had a lower odds of treatment at an HVC (AOR, 0.63; 95% CI, 0.47–0.86) than white patients with private insurance. Notably, white patients with Medicare insurance had odds of treatment at an HVC similar to the odds of those with private insurance (AOR, 0.93; 95% CI, 0.85–1.01). Hispanic individuals with “other” insurances including uninsured and unclassified insurance types had the lowest odds of receiving care at an HVC (AOR, 0.31 [95% CI, 0.20–0.48]; reference, white race, private insurance).

### Adjusted Trends

**Fig. 4:**
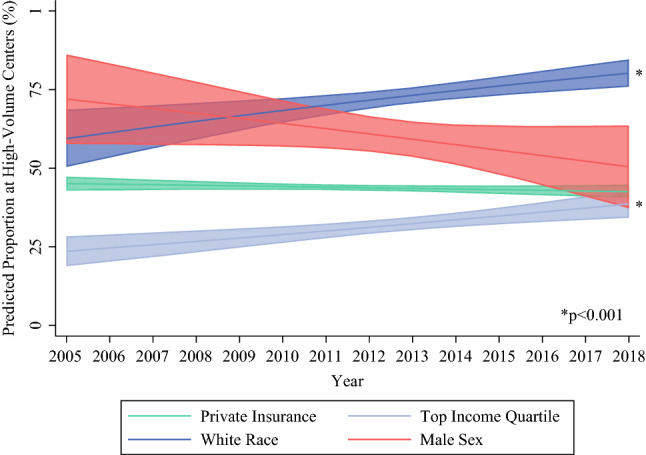
Risk-adjusted proportion of patients treated at high-volume centers by year

As shown in Fig. 4, proportions of patient demographics treated at an HVC were examined during the study period. The proportions of patients treated at an HVC who identified as male or as holding private insurance did not change during the study period. However, patients categorized as white or within the top income quartile increased in proportion to those treated at an HVC during the study period (Fig. 4).

## Discussion

In this nationally representative analysis, operative management of pancreatic malignancies was limited at HVCs for non-white, female, governmentally insured, and low-income populations. Furthermore, HVCs realized superior perioperative outcomes for this pancreatectomy cohort. Notably, racial differences in access to care persisted in subgroup analysis by insurance type. Surprisingly, these socioeconomic and demographic disparities did not improve during the study period, paradoxically worsening as those without private insurance and those of a non-white race were treated in decreasing proportions at HVCs over time.

Although decreased access to surgical care for minority groups has been noted in prior studies.^5,22^ the current study is the first to demonstrate the vast extent of access disparities in this operative population. These findings merit further discussion.

Disparities in access to high-quality surgical care have been noted across medical and surgical specialties,^5,8^ with surgical resection of malignancy serving as a common example of this phenomenon.^23^ However, the current study highlighted the full extent of these inequities, demonstrating that insurance status, income, biologic sex, and race all affect the ability of patients to receive treatment at an HVC.

The Social and Vulnerability Index as described by Hyer et al.^24^ combines many factors that may in part explain such inequities. This index uses housing, transportation, minority status, language, and socioeconomic status to categorize the social determinants of health for each patient. Using this index, the authors noted that community characteristics have an impact on health outcomes for pancreatectomy patients, which may in part explain our findings.^24^ Moreover, the current literature supports the notion that historically disadvantaged populations may require farther travel than others to reach high-quality surgical centers.^23,25^ Although this effect cannot be assessed by the current database, it may point to a major factor in persistent outcome disparities for those of a lower socioeconomic status.

A promising proposal to alleviate these health care inequities began with Medicaid expansion, which improved early cancer diagnoses and access to malignancy operations for patients of low socioeconomic status.^26^ Furthermore, investment in public surgical systems, focused patient education, and centralization of surgical mal-management of malignancies has been postulated to further improve these disparate outcomes on the large scale.^27^ Future investigation into these disparities and implementation of public policy to reduce these inequities are necessary to improve patient outcomes across demographic subgroups.

The focus on centralization of care has grown significantly in the United States since the implementation of the Leapfrog guidelines, which established minimum volume recommendations for major operations.^16^ Although the volume-outcome association has been well-established in surgical literature,^28,29^ its association with insurance, income, gender, and race is of particular concern.

In our study, historically marginalized patients experienced higher odds of mortality, complications, and non-routine discharge, likely secondary to hospital selection.^3^ Notably, high-volume and high-quality surgical centers have costs similar to those of lower-volume institutions, as noted in our investigation, emphasizing that once accessed, the baseline cost remains comparable. This finding supports the conclusion that the costs of cancer screening, primary care, referrals, and travel may be limiting factors for low-income patients rather than the cost of the operation itself.

Because HVCs may offer a higher level of care than LVCs but decreased access for historically marginalized communities, it is possible that a compromise may exist in a medium-volume center to maximize both outcomes and access. However, we are unable to comment on what constitutes an acceptable risk for pancreatic cancer resections at this time. Therefore, future studies examining a middle ground of quality and access may be warranted.

Despite increasing identification of surgical disparities and focused programs to improve outcomes such as the Affordable Care Act and the Leapfrog Initiative, our study found no improvement in access to HVCs among those with Medicaid insurance or among female patients undergoing pancreatectomy across the study era. Notably, the proportion of white patients and those within the highest income quartile treated at HVCs paradoxically increased during the study period. Importantly, in our study, black and Hispanic patients continued to have less access to HVCs than white patients despite their holding of similar insurance types.

Previous studies have shown that cancer survival has worsened for non-white patients during the past decade regardless of increasing national attention.^30,31^ The findings of these studies align with our results, which demonstrate increasing proportions of white patients treated at HVCs from 2005 to 2018. Several hypotheses may explain the reasons for this widening gap. First, early recognition of pancreatic malignancy is crucial to prolonged survival, which may be more achievable for those with established primary care physicians and higher levels of medical literacy.^32,33^ Second, findings have shown that modifiable risk factors such as decreased access to health care, increased alcohol consumption, higher rates of smoking, and greater incidence of vitamin D deficiency are more prominent in minority populations, likely due to under-treatment and limited resources.^34^ Finally, as routine screening guidelines and technologies improve, these changes may benefit those who have close follow-up assessment within the medical system, except for patients with more limited access.^31,35,36^ Further studies are warranted to identify the exact nature of these differences in order to increase the odds of survival for all patients afflicted with pancreatic cancer.

## Study Limitations

This study was limited by the administrative nature of the NIS and its available variables. Specific markers of clinical severity including lab data, imaging, and operative summaries were not captured by the NIS. Furthermore, this study spanned the ICD-9 and ICD-10 coding eras, which may have caused misclassifications of procedures and diagnoses due to coding conversions. Importantly, cancer staging, neoplasm size, chemotherapy history, radiation exposure, and nutritional parameters are not captured by the NIS. Moreover, the study population represented solely those treated with operative intervention rather than all-comers, restricting a full-access analysis. However, we anticipate that disparities in access may be even more pronounced than shown in this study because access to any operation may be limited in historically marginalized groups. Finally, the authors were unable to assess distance traveled to surgical centers, which may have affected hospital selection in this cohort. Despite these limitations, the current study offers a national, hospital-based perspective on disparate access to high-quality surgical care using the largest database of its type.

## Conclusions

Access to an HVC remains limited for those in historically disadvantaged groups despite the implementation of multiple policy changes during the study period. Moreover, HVCs continue to offer significantly better patient outcomes than LVCs. Ongoing assessment of factors contributing to access inequities and systematic approaches to alleviate these inequalities among the socioeconomically disadvantaged are necessary to improve outcomes for pancreatic malignancies.

## References

[1] Gooiker GA, Society on Behalf of the SCC of the DC, van Gijn W, et al. Systematic review and meta-analysis of the volume–outcome relationship in pancreatic surgery. *Br J Surg*. 2011;98:485–94. Doi:10.1002/BJS.7413. 10.1002/bjs.7413 21500187

[2] Rahib L, Smith BD, Aizenberg R, Rosenzweig AB, Fleshman JM, Matrisian LM (2014). Projecting cancer incidence and deaths to 2030: the unexpected burden of thyroid, liver, and pancreas cancers in the united states. Cancer Res..

[3] Stitzenberg KB, Sigurdson ER, Egleston BL, Starkey RB, Meropol NJ (2009). Centralization of cancer surgery: implications for patient access to optimal care. J Clin Oncol..

[4] Fonseca AL, Khan H, Mehari KR, Cherla D, Heslin MJ, Johnston FM (2022). Disparities in access to oncologic care in pancreatic cancer: a systematic review. Ann Surg Oncol..

[5] Nabi J, Tully KH, Cole AP (2021). Access denied: the relationship between patient insurance status and access to high‐volume hospitals. Cancer.

[6] Swords DS, Mulvihill SJ, Brooke BS, Skarda DE, Firpo MA, Scaife CL (2019). Disparities in utilization of treatment for clinical stage I-II pancreatic adenocarcinoma by area socioeconomic status and race/ethnicity. Surgery..

[7] Bilimoria KY, Bentrem DJ, Ko CY, Stewart AK, Winchester DP, Talamonti MS (2007). National failure to operate on early-stage pancreatic cancer. Ann Surg..

[8] Liu JH, Zingmond DS, McGory ML (2006). Disparities in the utilization of high-volume hospitals for complex surgery. JAMA..

[9] Singal V, Singal AK, Kuo YF (2012). Racial disparities in treatment for pancreatic cancer and impact on survival: a population-based analysis. J Cancer Res Clin Oncol..

[10] Gooiker GA, Lemmens VEPP, Besselink MG (2014). Impact of centralization of pancreatic cancer surgery on resection rates and survival. Br J Surg..

[11] Shapiro M, Chen Q, Huang Q (2016). Associations of socioeconomic variables with resection, stage, and survival in patients with early-stage pancreatic cancer. JAMA Surg..

[12] Epstein AJ, Gray BH, Schlesinger M (2010). Racial and ethnic differences in the use of high-volume hospitals and surgeons. Arch Surg..

[13] NIS Hospital Ownership Files. Retrieved 23 July 2020 at https://www.hcup-us.ahrq.gov/db/nation/nis/nisownership.jsp.

[14] Aguayo E, Antonios J, Sanaiha Y (2020). National trends in readmission and resource utilization after pancreatectomy in the United States. J Surg Res..

[15] Birkmeyer JD, Finlayson EVA, Birkmeyer CM (2001). Volume standards for high-risk surgical procedures: potential benefits of the Leapfrog initiative. Surgery..

[16] Hospital Ratings and Reports/Leapfrog. Retrieved 22 May 2022 at https://www.leapfroggroup.org/ratings-reports.

[17] Livingston EH, Cao J (2010). Procedure volume as a predictor of surgical outcomes. JAMA..

[18] Van Walraven C, Austin PC, Jennings A, Quan H, Forster AJ (2009). A modification of the elixhauser comorbidity measures into a point system for hospital death using administrative data. Med Care..

[19] Dunn A, Grosse SD, Zuvekas SH (2018). Adjusting health expenditures for inflation: a review of measures for health services research in the United States. Health Serv Res..

[20] Zou H, Hastie T (2005). Regularization and variable selection via the elastic net. J R Stat Soc Ser B Stat Methodol..

[21] Tibshirani R (1996). Regression shrinkage and selection via the Lasso. J R Stat Soc Ser B..

[22] Blanco BA, Kothari AN, Blackwell RH (2017). “Take the volume pledge” may result in disparity in access to care. Surgery..

[23] Nabi J, Tully KH, Cole AP (2021). Access denied: the relationship between patient insurance status and access to high-volume hospitals. Cancer..

[24] Hyer JM, Tsilimigras DI, Diaz A (2021). High social vulnerability and “textbook outcomes” after cancer operation. J Am Coll Surg..

[25] Wasif N, Etzioni D, Habermann EB (2018). Racial and socioeconomic differences in the use of high-volume commission on cancer-accredited hospitals for cancer surgery in the United States. Ann Surg Oncol..

[26] Mesquita-Neto JWB, Cmorej P, Mouzaihem H, Weaver D, Kim S, Macedo FI (2020). Disparities in access to cancer surgery after Medicaid expansion. Am J Surg..

[27] Sullivan R, Alatise OI, Anderson BO (2015). Global cancer surgery: delivering safe, affordable, and timely cancer surgery. Lancet Oncol..

[28] Gandjian M, Williamson C, Sanaiha Y (2021). Continued relevance of minimum volume standards for elective esophagectomy: a national perspective. Ann Thorac Surg..

[29] Finks JF, Osborne NH, Birkmeyer JD (2011). Trends in hospital volume and operative mortality for high-risk surgery. N Engl J Med..

[30] Terplan M, Schluterman N, McNamara EJ, Tracy JK, Temkin SM (2012). Have racial disparities in ovarian cancer increased over time? An analysis of SEER data. Gynecol Oncol..

[31] Corona E, Yang L, Esrailian E, Ghassemi KA, Conklin JL, May FP (2021). Trends in esophageal cancer mortality and stage at diagnosis by race and ethnicity in the United States. Cancer Causes Control..

[32] Paasche-Orlow MK, Wolf MS (2010). Promoting health literacy research to reduce health disparities. J Health Commun.

[33] Rose PW, Rubin G, Perera-Salazar R (2015). Explaining variation in cancer survival between 11 jurisdictions in the International Cancer Benchmarking Partnership: a primary care vignette survey. BMJ Open..

[34] Rawla P, Sunkara T, Gaduputi V (2019). Epidemiology of pancreatic cancer: global trends, etiology, and risk factors. World J Oncol..

[35] Wilder JM, Oloruntoba OO, Muir AJ, Moylan CA (2016). Role of patient factors, preferences, and distrust in health care and access to liver transplantation and organ donation. Liver Transplant..

[36] Jerant AF, Fenton JJ, Franks P (2008). Determinants of racial/ethnic colorectal cancer screening disparities. Arch Intern Med..

